# Berberine as a multitarget modulator of metabolic inflammation: molecular mechanisms, circadian regulation, and translational perspectives

**DOI:** 10.3389/fphar.2026.1935151

**Published:** 2026-09-08

**Authors:** Jiaxuan Xie, Junbo Cao, Shuying Zhang, Jiayang Zhang, Guanlin Wu, Xinyu Qiu, Lihong Chen, Guangrui Yang

**Affiliations:** 1 Graduate School, Shanghai University of Traditional Chinese Medicine, Shanghai, China; 2 School of Basic Medical Sciences, Shanghai University of Medicine and Health Sciences, Shanghai, China; 3 School of Clinical Medicine, Shanghai University of Medicine and Health Sciences, Shanghai, China; 4 Health Science Center, East China Normal University, Shanghai, China

**Keywords:** berberine, circadian rhythm, clinical translation, gut microbiota, metabolic inflammation, mitochondrial homeostasis

## Abstract

Metabolic inflammation is a chronic, low-grade inflammatory state sustained by metabolic stress, organelle dysfunction, immune activation, and gut-derived signals. Because these processes are closely interconnected, single-target interventions may be insufficient. Berberine, a plant-derived isoquinoline alkaloid, has attracted attention for its broad metabolic and anti-inflammatory effects. In this review, we summarize evidence that berberine regulates several interacting pathways, including cellular energy sensing, glucose and lipid metabolism, mitochondrial quality control, redox and inflammatory signaling, and gut–liver–microbiota communication. We also discuss circadian regulation as a relevant but still underexplored aspect of berberine action, focusing on BMAL1/CLOCK- and REV-ERBα-related metabolic and inflammatory networks. Current clinical evidence suggests modest benefits for glycemic control, insulin resistance, dyslipidemia, and selected features of steatotic liver disease. However, formulation variability, heterogeneous trial designs, and limited long-term outcome data remain important barriers. Overall, berberine may serve as an adjunctive metabolic modulator, but standardized preparations and time-aware clinical studies are needed.

## Graphical Abstract

**Figure:**
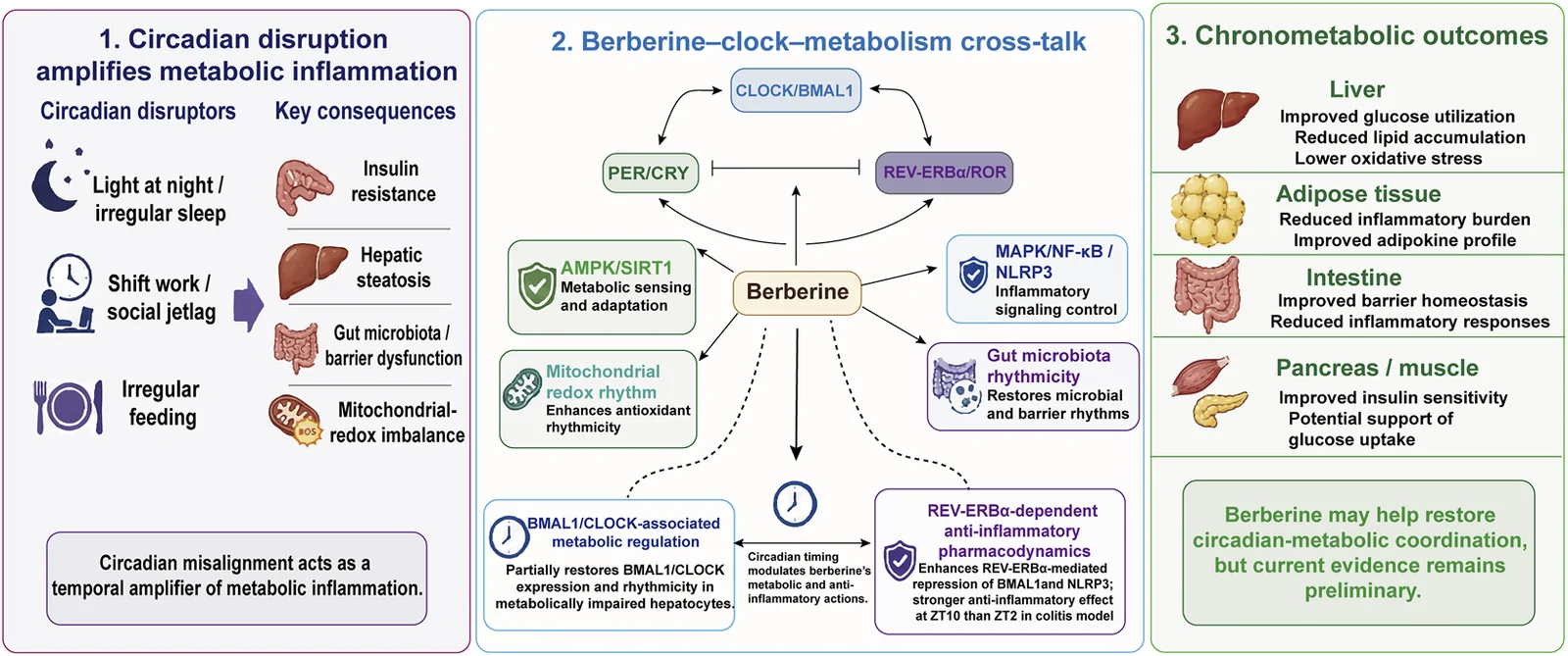

## 1 Introduction

Metabolic disorders encompass a clinically heterogeneous group of diseases that share a common pathological substrate. This chronic, low-grade, sterile inflammatory state arises when sustained nutrient and metabolic stress exceed the adaptive capacity of metabolic tissues (Gaber et al., 2017). Lipotoxicity, glucotoxicity, mitochondrial and endoplasmic reticulum stress, intestinal barrier dysfunction, and maladaptive immune activation then reinforce one another, progressively disrupting insulin action, lipid handling, vascular homeostasis, and endocrine function (Galassetti, 2012). Current therapies have substantially improved the control of glucose, lipids, body weight, and cardiovascular risk. Nevertheless, residual inflammatory risk, heterogeneous treatment responses, poor long-term adherence, and progressive multi-organ injury remain important clinical challenges (Roth et al., 2002; Prasad et al., 2012; Kao and Huang, 2021). Because metabolic inflammation is maintained by mutually reinforcing networks rather than a single dominant lesion, agents capable of modulating several interconnected pathological nodes may provide complementary therapeutic value (Suren Garg et al., 2023).

Berberine (BBR) is a protoberberine isoquinoline alkaloid derived from Coptidis Rhizoma, Phellodendri Chinensis Cortex, Berberis species, and other medicinal plants (Imenshahidi and Hosseinzadeh, 2016). It has a long history of use in traditional Chinese medicine, particularly for gastrointestinal infections, diarrhea, and inflammatory disorders. Modern pharmacological studies have expanded its therapeutic profile, demonstrating glucose-lowering, lipid-regulating, anti-inflammatory, antioxidant, hepatoprotective, cardioprotective, neuroprotective, and anticancer activities (Hou et al., 2020; Song et al., 2020). These pleiotropic effects are attributed to its ability to modulate multiple signaling pathways involved in energy metabolism, oxidative stress, immune responses, and cell survival (Plazas et al., 2022). Despite its relatively poor oral bioavailability, BBR can achieve extensive tissue distribution and generate pharmacologically active metabolites, which may partly account for its broad *in vivo* efficacy (Och et al., 2022). Collectively, these properties have positioned BBR as a promising multitarget natural compound for the prevention and treatment of metabolic and inflammation-related diseases.

Previous reviews have provided broad summaries of BBR pharmacology and clinical evidence across individual metabolic diseases or have focused specifically on pharmacokinetic and bioavailability barriers (Xu et al., 2021; Cui et al., 2024). Building on, but distinct from, these approaches, this review aims to reinterpret BBR as a multitarget modulator of metabolic inflammation by organizing the evidence around metabolic inflammation as an inter-organ and time-dependent network rather than a catalogue of diseases or isolated signaling pathways. We first define this network as being driven by nutrient overload, organelle stress, gut-derived signals, immune activation, and circadian misalignment, and then synthesize evidence that BBR acts across five interacting mechanistic hubs: mitochondrial energy sensing, AMPK/SIRT1/PGC-1α-dependent adaptation, glucose and lipid metabolic reprogramming, inflammatory and redox network regulation, and gut microbiota–intestinal barrier–bile acid signaling. Within this network model, we further place circadian regulation across these hubs as a temporal organizing layer spanning feeding–fasting cycles, tissue clocks, microbial rhythms, and inflammatory responsiveness. We also distinguish gut-localized pharmacology from systemic target engagement and connect formulation-dependent exposure with disease-specific clinical translation. Finally, we evaluate disease-specific clinical evidence, formulation and safety barriers, and future strategies for precision and chronometabolic translation. Together, this framework is intended to clarify when, where, and under what pharmacokinetic context the multitarget actions of BBR may become clinically meaningful.

## 2 Ethnopharmacological and pharmacokinetic basis of berberine

### 2.1 Traditional origins and chemical characteristics

Berberine is a naturally occurring isoquinoline alkaloid and one of the major bioactive components of several medicinal plants. Its most representative source is Coptidis Rhizoma, the dried rhizome of *Coptis chinensis* Franch., *Coptis deltoidea* C. Y. Cheng et Hsiao, and *Coptis teeta* Wall. Other important sources include Phellodendri Chinensis Cortex, *Berberis vulgaris* L., *Berberis aristata* DC., *Hydrastis canadensis* L., *Mahonia aquifolium*, and related alkaloid-rich plants. Pharmacognostic and phytochemical analyses of Berberis aristata DC. roots further support this species as an alkaloid-rich botanical source containing berberine (Jain et al., 2023b). In traditional Chinese medicine, Coptidis Rhizoma is characterized as bitter and cold, with the functions of clearing heat, drying dampness, purging fire, and detoxifying (Hou et al., 2020). These traditional indications are consistent with its long-standing use in intestinal infection, diarrhea, inflammatory conditions, and disorders now understood to involve metabolic and immune dysregulation (Imenshahidi and Hosseinzadeh, 2016). Chemically, berberine (BBR) belongs to the protoberberine alkaloid family and possesses a rigid, planar tetracyclic scaffold with a quaternary ammonium center. Its extended conjugated structure contributes to its characteristic yellow color and facilitates interactions with diverse biological macromolecules, whereas its permanent positive charge and limited lipophilicity restrict passive membrane permeability and oral absorption (Luo et al., 2024; Albukhari, 2026). The methylenedioxy moiety represents an important structural determinant of its enzyme-binding properties. *In vitro*, BBR strongly inhibited CYP1B1, whereas CYP1A2 was comparatively resistant. CYP1A1- and CYP1B1-mediated oxidation primarily generated demethyleneberberine, with smaller amounts of thalifendine. Oxidative preincubation did not enhance CYP1 inhibition, indicating minimal metabolism-dependent irreversible inactivation. Moreover, ring-opened or demethylated derivatives, including demethyleneberberine, thalifendine, berberrubine, and jatrorrhizine, together with structurally related protoberberines such as palmatine, exhibited weaker CYP1 inhibition than the parent compound (Lo et al., 2015; Cui et al., 2024). These observations suggest that the intact methylenedioxy group contributes to the potency and selectivity of BBR toward CYP1B1, whereas oxidative modification generally attenuates this activity. The same structural features that support its pharmacological activity also partly explain its unfavorable pharmacokinetic behavior. Therefore, the chemical nature of BBR should not be regarded only as a structural description, but as a key determinant of its absorption, distribution, metabolism, target engagement, and formulation challenges.

### 2.2 Pharmacokinetics, metabolism, and the bioavailability paradox

A central translational issue for BBR is its poor systemic bioavailability. After oral administration, BBR is limited by low solubility, poor intestinal permeability, P-glycoprotein-mediated efflux, and extensive intestinal and hepatic metabolism (Och et al., 2020). Absolute oral bioavailability in rodents is generally below 1%, and very low systemic exposure has also been reported in human pharmacokinetic studies; for example, mean peak plasma concentrations were approximately 0.4 ng/mL after 400 mg oral BBR in one study, whereas another study reported a Cmax of only 0.07 ± 0.01 nM after a single 500 mg dose (Xu et al., 2021; Cui et al., 2024). BBR can nevertheless be converted into several metabolites, including demethyleneberberine, berberrubine, thalifendine, jatrorrhizine, and their glucuronidated or sulfated derivatives. Notably, several metabolites retain substantial pharmacological activity; for example, berberrubine and palmatine enhance GLP-1 secretion by protecting intestinal L cells from inflammatory, oxidative, and mitochondrial injury (Yang et al., 2024). The low plasma exposure of BBR therefore contrasts with its broad pharmacological efficacy, forming a “bioavailability paradox.” This paradox is particularly relevant to metabolic inflammation because the intestine and liver are not only sites of drug absorption and metabolism but also central organs in the initiation and amplification of metabolic inflammation. High local intestinal exposure, enterohepatic circulation, microbial transformation, bile acid modulation, active metabolites, and hepatic distribution may allow BBR to exert meaningful biological effects even when circulating concentrations of the parent compound are low (Liu et al., 2022).

At the same time, poor bioavailability remains a real barrier for clinical translation. Strategies including structural modification, absorption enhancers, P-glycoprotein inhibitors, salts, cocrystals, nanoparticles, liposomes, phospholipid complexes, and other delivery systems have therefore been explored (Cui et al., 2024). Recent studies illustrate both the magnitude and the diversity of formulation effects. A Gantrez–phospholipid lipid–polymer hybrid nanoparticle formulation (BER-LPHN) increased Cmax from 3.18 to 22.14 μg/mL and AUC0–24 from 12.80 to 242.04 μg h/mL after oral administration in rats, corresponding to an approximately 18.9-fold increase in relative bioavailability; Peyer’s patch uptake and lymphatic transport were implicated in partial avoidance of first-pass metabolism (Thool and Pokharkar, 2025). A carrier-free BBR–magnolol nanoassembly increased serum Cmax by approximately 3.8-fold and AUC0–t by 3.24-fold while also increasing colonic BBR accumulation (Xu Y. et al., 2024), showing that nanoassembly can alter both systemic exposure and tissue distribution. For local delivery, a berberine-loaded silk fibroin/polycaprolactone electrospun nanofibrous membrane (Ber@SF/PCL) provided an initial release followed by sustained local delivery and enhanced alveolar bone regeneration in diabetic rats (Ming et al., 2024), illustrating that formulation optimization may also aim to increase exposure at the therapeutic site rather than plasma exposure alone. Beyond nanocarriers, recent crystal engineering generated a berberine–gentisic acid salt with improved solubility and permeability and an approximately 1.8-fold higher peak plasma concentration than berberine chloride (Surampudi et al., 2025).

The clinical relevance of improved bioavailability therefore depends on the intended site and mechanism of action. For indications requiring systemic target engagement, higher and more reproducible exposure could potentially reduce the oral dose needed to reach effective concentrations, lessen dose-related gastrointestinal burden, reduce interindividual pharmacokinetic variability, improve access to extraintestinal targets, and make dose–response and drug–interaction assessment more interpretable. These advantages, however, remain translational expectations rather than established clinical benefits for most enhanced-bioavailability BBR products. Increasing plasma exposure may change the contribution of beneficial gut-localized actions, alter metabolite profiles, or introduce systemic safety liabilities. Formulation success should therefore be judged by indication-specific pharmacokinetic–pharmacodynamic relationships, tolerability, and clinically meaningful outcomes rather than by Cmax or AUC alone.

### 2.3 Multitarget pharmacological profile relevant to metabolic inflammation

BBR influences mitochondrial function, oxidative stress, autophagy, and cellular stress responses. Mitochondrial dysfunction is a key amplifier of metabolic inflammation, and BBR-mediated improvement of mitochondrial-redox homeostasis may contribute to its protective effects in the liver, adipose tissue, vascular system, and pancreas. The convergence of AMPK activation, inflammatory suppression, gut microbiota modulation, and mitochondrial protection provides the mechanistic basis for considering BBR as a multitarget modulator rather than a single-pathway intervention (Song et al., 2020). An overview of the ethnopharmacological origins, pharmacological characteristics, formulation strategies, and metabolic applications of BBR is presented.

## 3 Metabolic inflammation: pathological basis

### 3.1 From metabolic overload to chronic immunometabolic dysfunction

Metabolic inflammation is a chronic, low-grade, sterile inflammatory state induced by sustained metabolic stress rather than microbial infection. It develops when nutrient availability persistently exceeds the storage, processing, and oxidative capacity of metabolic tissues, leading to glucotoxicity, lipotoxicity, organelle stress, and maladaptive immune activation (Masenga et al., 2023).

Excess fatty acids, ceramides, cholesterol crystals, advanced glycation products, oxidized lipids, extracellular ATP, and damage-associated molecules released from stressed cells activate inflammatory sensing pathways in both metabolic and immune cells (Xu S. et al., 2024). The resulting cytokine and chemokine production impairs insulin signaling, nutrient utilization, and tissue repair, thereby generating further metabolic stress. Metabolic inflammation is therefore characterized by reciprocal reinforcement between metabolic dysfunction and immune activation.

Insulin resistance is a central consequence and amplifier of this process. Reduced insulin responsiveness increases hepatic glucose production, limits peripheral glucose uptake, and promotes adipose-tissue lipolysis. The resulting elevations in circulating glucose and free fatty acids further expose the liver, skeletal muscle, pancreas, and vasculature to metabolic overload. This self-sustaining interaction provides a common pathological basis for obesity, type 2 diabetes mellitus, metabolic dysfunction-associated steatotic liver disease, dyslipidemia, and cardiovascular disease.

### 3.2 Tissue-specific origins and inter-organ propagation

Adipose tissue is often an early site of metabolic inflammation. During chronic energy excess, adipocyte hypertrophy is accompanied by local hypoxia, extracellular-matrix remodeling, altered adipokine secretion, and immune-cell recruitment. These changes reduce insulin sensitivity and accelerate lipolysis, increasing the delivery of non-esterified fatty acids to other metabolic organs (Roche, 2019).

The liver integrates dietary nutrients, adipose-derived fatty acids, hormonal signals, and gut-derived metabolites. When fatty acid influx and *de novo* lipogenesis exceed oxidation and lipid export, hepatic steatosis develops. Prolonged lipid accumulation generates lipotoxic intermediates and hepatocellular stress, promoting macrophage activation and stellate-cell responses that drive progression toward steatohepatitis and fibrosis.

Metabolic stress also disrupts glucose utilization in skeletal muscle and compromises compensatory insulin secretion in pancreatic β-cells. In the vascular system, endothelial dysfunction, oxidized lipoprotein retention, and monocyte recruitment create a pro-atherogenic environment. Thus, metabolic inflammation propagates through circulating substrates, cytokines, hormones, and microbial products, converting initially localized tissue dysfunction into systemic disease.

### 3.3 Organelle stress, redox imbalance, and loss of metabolic flexibility

Mitochondria and the endoplasmic reticulum are major intracellular sensors of nutrient overload. Excess substrate supply increases electron leakage from the respiratory chain and promotes reactive oxygen species production. When antioxidant defenses become insufficient, oxidative damage impairs mitochondrial DNA, respiratory proteins, and membrane lipids, further reducing metabolic efficiency and increasing cellular stress.

Defective mitochondrial quality control, including impaired mitophagy and disturbed fission–fusion balance, allows damaged mitochondria to accumulate. This limits fatty acid oxidation and metabolic flexibility while increasing the release of mitochondrial danger signals that sustain innate immune activation.

Endoplasmic reticulum stress develops concurrently as altered lipid composition, increased protein synthesis, and disturbed calcium homeostasis impair protein folding. Although the unfolded protein response is initially adaptive, persistent activation promotes inflammatory signaling, insulin resistance, and cell death. Mitochondrial dysfunction, oxidative stress, and endoplasmic reticulum stress therefore form an interconnected network that links nutrient excess to chronic metabolic inflammation (Yuan et al., 2022).

### 3.4 Gut-derived signals and systemic metabolic inflammation

The intestinal ecosystem provides an additional route through which metabolic stress is amplified. Diet, medication exposure, aging, and metabolic disease can alter microbial functions and compromise epithelial barrier integrity. Increased permeability facilitates the entry of lipopolysaccharide and other microbial products into the portal and systemic circulation, intensifying hepatic and systemic inflammatory responses (Boulangé et al., 2016).

Microbial metabolites also influence host metabolism. Short-chain fatty acids generally support epithelial integrity, enteroendocrine signaling, and immune regulation, whereas altered bile acid metabolism and other unfavorable microbial products may contribute to insulin resistance, hepatic steatosis, and vascular dysfunction. The pathological significance of the gut microbiota should therefore be interpreted in terms of microbial function, metabolite production, and barrier integrity rather than individual taxa or simple phylum-level ratios.

Circadian disruption may further amplify these processes. Feeding behavior, hormone secretion, mitochondrial metabolism, intestinal permeability, microbial activity, and bile acid turnover are temporally regulated. Irregular eating, sleep restriction, and light exposure can therefore uncouple behavioral rhythms from peripheral metabolic clocks, aggravating metabolic and inflammatory dysfunction. Together, nutrient overload, organelle stress, gut-derived signals, and circadian misalignment establish a self-reinforcing pathological environment that supports the development and progression of metabolic disease.

### 3.5 Circadian disruption as a temporal amplifier of metabolic inflammation

Metabolic inflammation is not only spatially distributed across organs but also temporally organized. Feeding–fasting cycles, sleep–wake behavior, hormone secretion, mitochondrial substrate utilization, intestinal permeability, microbial activity, bile acid turnover, and immune responsiveness all display circadian rhythmicity. Under physiological conditions, the central clock in the suprachiasmatic nucleus and peripheral clocks in the liver, intestine, adipose tissue, skeletal muscle, pancreas, vasculature, and immune cells coordinate metabolic flux with predictable environmental and behavioral cycles.

Circadian disruption can amplify metabolic inflammation by uncoupling nutrient supply from tissue metabolic capacity. Irregular feeding, sleep restriction, shift work, inappropriate light exposure, and social jetlag disturb the alignment between behavioral rhythms and peripheral metabolic clocks. This misalignment impairs glucose tolerance, lipid handling, mitochondrial adaptation, redox balance, intestinal barrier function, bile acid signaling, and inflammatory resolution. The liver and gastrointestinal tract are particularly sensitive to these disturbances because their functions must be synchronized with recurring nutrient intake and microbial metabolic activity.

Importantly, the relationship between circadian disruption and metabolic inflammation is bidirectional. Nutrient excess, insulin resistance, hepatic steatosis, gut dysbiosis, and chronic cytokine exposure can weaken clock-gene oscillation and reduce rhythmic amplitude, whereas impaired circadian organization further aggravates metabolic stress and inflammatory signaling. Circadian dysregulation should therefore be regarded as a temporal amplifier of metabolic inflammation.

## 4 Multitarget mechanisms of berberine in ameliorating metabolic inflammation

The mechanisms of BBR should be interpreted as an interconnected regulatory network rather than a linear cascade. Mitochondrial complex I modulation and AMPK activation provide an upstream energy-sensing entry point, but their downstream consequences depend on tissue metabolic state, nutrient availability, inflammatory tone, intestinal exposure, and circadian timing. AMPK/SIRT1/PGC-1α signaling links energy stress to mitochondrial biogenesis, mitophagy, fatty acid oxidation, and antioxidant defense. Glucose and lipid metabolic reprogramming determines whether substrates are stored, oxidized, or exported. MAPK/NF-κB/NLRP3 and Nrf2/SIRT signaling integrate inflammatory and redox responses. Meanwhile, the gut microbiota, intestinal barrier, and bile acid pool connect local intestinal exposure to systemic metabolic effects.

**FIGURE 1:**
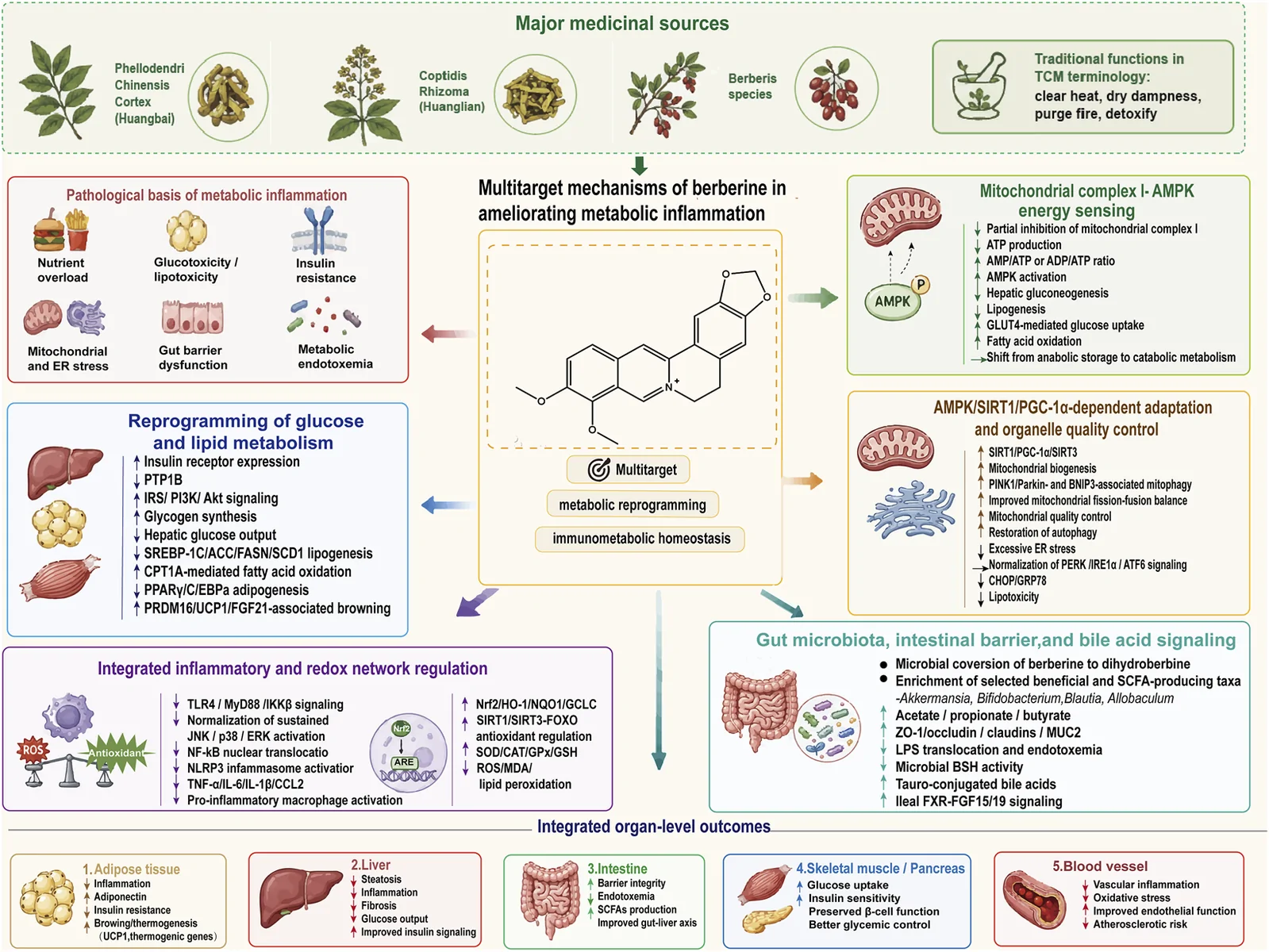
Multitarget mechanisms of berberine in ameliorating metabolic inflammation. Berberine is mainly derived from Coptidis Rhizoma, Phellodendri Chinensis Cortex, and Berberis species. Berberine alleviates metabolic inflammation through multiple interconnected mechanisms, including mitochondrial complex I–AMPK energy sensing, AMPK/SIRT1/PGC-1α-dependent organelle adaptation and quality control, reprogramming of glucose and lipid metabolism, integrated inflammatory and redox network regulation, and gut microbiota–intestinal barrier–bile acid signaling. Collectively, these effects improve metabolic homeostasis and confer multi-organ protection in adipose tissue, liver, intestine, skeletal muscle/pancreas, and vasculature.

These hubs interact bidirectionally. For example, mitochondrial ROS can activate inflammatory signaling, whereas inflammation impairs insulin action and mitochondrial quality control. Gut-derived LPS can stimulate hepatic and adipose inflammation, whereas bile acid and SCFA signaling can reshape glucose, lipid, and immune responses. Circadian clocks further gate the timing of mitochondrial activity, microbial metabolism, bile acid turnover, and inflammatory responsiveness. Thus, BBR is best understood as a multitarget metabolic recalibrator that shifts an inflamed, substrate-overloaded network toward improved metabolic flexibility and immunometabolic homeostasis. Figure 1 summarizes this network model.

### 4.1 Mitochondrial energy sensing as an upstream trigger of berberine action

AMP-activated protein kinase is widely regarded as a central mediator of the metabolic effects of berberine. However, describing berberine simply as a direct AMPK activator may overlook the upstream bioenergetic events that initiate this response. Berberine can partially inhibit mitochondrial respiratory-chain complex I, reduce oxidative phosphorylation and ATP production, and increase the AMP/ATP or ADP/ATP ratio (Yu et al., 2021). This energetic shift subsequently activates AMPK and redirects metabolism from energy-consuming anabolic processes toward catabolic and stress-adaptive programs.

In the liver, AMPK activation suppresses gluconeogenesis and *de novo* lipogenesis while promoting fatty acid utilization. In adipose tissue and skeletal muscle, it enhances glucose uptake, substrate oxidation, and insulin responsiveness. In immune and vascular cells, AMPK restrains inflammatory activation and oxidative stress. Complex I modulation and AMPK activation should therefore be interpreted as sequential components of a mitochondrial energy-sensing response rather than as unrelated mechanisms. Importantly, partial complex I inhibition does not necessarily indicate generalized mitochondrial toxicity. Under conditions of excessive nutrient supply, moderate respiratory restraint may reduce substrate overload, limit anabolic lipid flux, and initiate adaptive metabolic reprogramming. Experimental studies have associated berberine-induced respiratory inhibition in the intestine and liver with reduced nutrient absorption, altered lipid handling, and improvement of obesity or hepatic steatosis (Wang et al., 2017).

BBR-induced mitochondrial complex I modulation can be therapeutically adaptive under nutrient-overloaded conditions because it reduces energetic excess, activates AMPK, and redirects metabolism toward substrate utilization. This benefit is most plausible within a moderate exposure range; excessive or prolonged respiratory inhibition may become detrimental in tissues with severe pre-existing mitochondrial failure. In nutrient-overloaded tissues, controlled respiratory restraint may be beneficial, whereas in tissues with established mitochondrial failure, berberine may promote mitochondrial biogenesis, substrate oxidation, and functional recovery (He et al., 2025). At higher concentrations or prolonged exposure, excessive respiratory inhibition could instead cause ATP depletion or cellular injury. Berberine may therefore function as a context-dependent mitochondrial recalibrator rather than uniformly enhancing or suppressing mitochondrial activity.

### 4.2 AMPK/SIRT1/PGC-1α-dependent adaptation and organelle quality control

Following cellular energy sensing, the AMPK/SIRT1/PGC-1α axis coordinates many of the adaptive responses induced by berberine. AMPK and SIRT1 interact through changes in cellular NAD^+^ metabolism and reciprocal regulation of downstream substrates. Together, they influence PGC-1α, a transcriptional coactivator involved in mitochondrial biogenesis, fatty acid oxidation, oxidative metabolism, and antioxidant defense (Qin et al., 2020).

This axis is particularly relevant in tissues with high metabolic demand. In metabolically stressed hepatic, renal, cardiac, and skeletal muscle cells, restoration of PGC-1α-associated programs may improve mitochondrial bioenergetics, substrate utilization, and metabolic flexibility. These observations suggest that the mitochondrial effects of berberine extend beyond changes in ATP production and include recovery of tissue-specific energy metabolism (Alpaslan Ağaçdiken and Göktaş, 2025).

Berberine also affects mitochondrial turnover. PINK1/Parkin-, BNIP3-, and related mitophagy pathways have been implicated in the selective removal of damaged mitochondria, while regulation of mitochondrial fusion and fission may help preserve organelle structure and function. By limiting the accumulation of dysfunctional mitochondria, berberine may reduce mitochondrial reactive oxygen species and organelle-derived danger signals that otherwise sustain inflammasome activation.

Mitochondrial biogenesis and mitophagy must remain coordinated. Increasing mitochondrial abundance without removing damaged organelles may aggravate oxidative stress, whereas excessive mitophagy without compensatory biogenesis may compromise energy supply. The benefits of berberine may therefore reflect restoration of mitochondrial turnover and quality rather than a simple increase in mitochondrial number or autophagic activity.

Endoplasmic reticulum stress and autophagy form an additional component of this adaptive response. Prolonged activation of PERK-, IRE1α-, and ATF6-associated unfolded-protein responses contributes to insulin resistance, inflammatory signaling, and cell death. Through AMPK activation and context-dependent regulation of mTOR, berberine may attenuate excessive endoplasmic reticulum stress and restore autophagic degradation of damaged organelles and lipid droplets.

### 4.3 Reprogramming of glucose and lipid metabolism

Insulin resistance is a major convergence point between nutrient overload and chronic inflammation. Berberine may improve insulin responsiveness by increasing insulin receptor expression, inhibiting protein tyrosine phosphatase 1B, and enhancing IRS/PI3K/Akt-associated signaling. Improved Akt activity promotes glucose uptake and glycogen synthesis, whereas modulation of GSK-3 and mTOR links insulin action with nutrient sensing, protein synthesis, and autophagy.

The direction of these effects is context-dependent. In insulin-resistant tissues, restoration of PI3K/Akt signaling can enhance glucose transport and glycogen storage. Under conditions of nutrient excess and suppressed autophagy, however, restraint of excessive mTOR activity may be advantageous. Berberine therefore appears to normalize signaling toward a physiologically appropriate state rather than uniformly activating or inhibiting the entire pathway (Zhou et al., 2025).

Berberine also reduces hepatic glucose production. AMPK-associated inhibition of gluconeogenic transcription, changes in cellular energy status, and modulation of glucagon-responsive pathways contribute to reduced expression or activity of key gluconeogenic enzymes. In peripheral tissues, increased glucose-transporter activity and glycolytic utilization may further improve glucose disposal. Intestinal inhibition of carbohydrate digestion and modulation of incretin-related signaling provide additional, predominantly local mechanisms (Zhang et al., 2026).

The effects of berberine on lipid metabolism encompass both reduced lipid storage and increased lipid utilization. In the liver, the AMPK–SREBP-1c–SCD1 axis represents a well-supported mechanism linking cellular energy status to lipogenic transcription (Zhu et al., 2019). Berberine can also influence ACC, FASN, CD36, FABP1, CPT1A, microsomal triglyceride-transfer pathways, and cholesterol transport. The resulting reduction in hepatic steatosis should therefore not be attributed to one enzyme, but to coordinated changes in fatty acid uptake, synthesis, esterification, oxidation, and export.

In adipose tissue, berberine may inhibit adipocyte differentiation through regulation of PPARγ, C/EBPα, and associated lipogenic programs. It has also been linked to mitochondrial biogenesis, lipolysis, thermogenic activation, and white-adipose browning through PGC-1α, PRDM16, UCP1, FGF21, and related factors. Growth differentiation factor 15 and central or enteroendocrine signals may further influence appetite and whole-body energy balance (Wang et al., 2021).

These findings extend the metabolic actions of berberine beyond inhibition of adipogenesis. They suggest a broader shift from nutrient storage toward utilization and energy expenditure. Nevertheless, most evidence for adipose browning and thermogenesis remains preclinical, and the extent to which these mechanisms account for weight reduction in humans remains uncertain.

### 4.4 Integrated inflammatory and redox network regulation

MAPKs, NF-κB, and the NLRP3 inflammasome form an interconnected inflammatory circuit linking nutrient excess, endotoxin, oxidative stress, and organelle damage to cytokine production. Persistent activation of JNK, p38, and ERK promotes inhibitory phosphorylation of insulin-signaling components, inflammatory transcription, macrophage activation, and tissue injury.

BBR primarily restrains sustained stress-activated MAPK signaling, especially JNK, p38, and ERK activation linked to insulin resistance and inflammatory injury. Rather than globally inhibiting MAPKs, BBR appears to normalize excessive inflammatory MAPK tone while preserving adaptive signaling required for cell survival. Transient activation of particular MAPK branches may participate in antioxidant adaptation or cell survival, whereas sustained activation contributes to insulin resistance and inflammatory injury. It is therefore more accurate to state that berberine normalizes dysregulated MAPK signaling in a stimulus-, tissue-, and dose-dependent manner.

NF-κB acts downstream of cytokine receptors, TLR4/MyD88 signaling, mitochondrial reactive oxygen species, and endoplasmic reticulum stress. Berberine can inhibit IKKβ activation, preserve IκBα, reduce nuclear translocation of NF-κB subunits, and decrease transcription of TNF-α, IL-6, IL-1β, CCL2, COX-2, iNOS, and related mediators. IL-6 is a pleiotropic pro-inflammatory cytokine with a well-established role in sustaining chronic inflammatory signaling (Mishra et al., 2024). In several models, this effect is linked to AMPK activation, illustrating how cellular energy sensing and inflammatory transcription are functionally coupled.

The NLRP3 inflammasome provides a further connection between organelle stress and inflammatory cytokine maturation. Mitochondrial reactive oxygen species, oxidized mitochondrial DNA, cholesterol crystals, lysosomal damage, and extracellular ATP can promote inflammasome assembly and caspase-1-dependent maturation of IL-1β and IL-18. Berberine may restrain this process by reducing NF-κB-dependent priming, restoring mitochondrial quality control, and limiting organelle-derived danger signals. Current evidence supports NLRP3 suppression as an important downstream anti-inflammatory outcome of BBR, mainly through reduced NF-κB priming, mitochondrial ROS, and organelle-derived danger signals. Direct molecular engagement of NLRP3 requires further validation.

Redox regulation occurs in parallel through reduced oxidant generation and reinforcement of endogenous antioxidant defenses. Berberine has been associated with activation of Nrf2-dependent transcription and increased expression of HO-1, NQO1, GCLC, superoxide dismutase, catalase, and glutathione-related enzymes. SIRT1 and SIRT3 further connect redox control with PGC-1α, FOXO proteins, mitochondrial enzymes, and NF-κB.Consistent with this broader anti-inflammatory and antioxidant profile, berberine isolated from Berberis aristata showed radical-scavenging activity *in vitro* and reduced arthritic severity and inflammatory biomarkers in a complete Freund’s adjuvant-induced arthritis model (Jain et al., 2023a). Although this is not a metabolic-disease model, it provides independent *in vivo* evidence for the redox- and inflammation-modulating pharmacology of BBR.

Evidence suggests that BBR’s antioxidant and anti-inflammatory activities are functionally coupled rather than independent. In LPS/macrophage and neurological models, Nrf2 activation by BBR simultaneously lowered ROS/NO and inflammatory mediators such as IL-6, IL-1β, and TNF-α; Nrf2 silencing weakened these protective effects, supporting a shared redox–inflammatory node rather than simple biomarker coexistence (Ashrafizadeh et al., 2020). The diabetic encephalopathy study further places mitochondrial ROS downstream of Rho/ROCK, suggesting that BBR can intercept upstream stress signals that feed inflammatory amplification. However, this relationship is context-dependent: in activated hepatic stellate cells, BBR increased ROS to drive ferroptosis and attenuate fibrosis (Yi et al., 2021). Thus, in metabolic inflammation, BBR is better viewed as a redox-state recalibrator that suppresses maladaptive oxidative–inflammatory coupling while permitting pro-oxidant signaling when therapeutically advantageous. Most studies, however, do not report formal correlation or mediation analyses, so causality between antioxidant changes and inflammatory biomarker reduction remains incompletely established.

The redox activity of berberine is not uniform across biological contexts. In nonmalignant metabolically stressed tissues, it generally reduces oxidative injury and protects mitochondrial function. In tumor cells or at higher concentrations, it may instead increase reactive oxygen species and promote apoptosis. This dual behavior further emphasizes that berberine pharmacology depends on baseline redox state, cellular metabolism, dose, and exposure duration.

Recent single-cell and multi-omics studies support the idea that berberine influences metabolic and inflammatory networks across multiple cell populations. However, changes in macrophage, neutrophil, fibroblast, or epithelial states observed in a particular disease model should not automatically be generalized to all metabolic disorders. Such findings are most appropriately interpreted as tissue-specific evidence of network remodeling rather than proof of universal immunological effects.

### 4.5 Gut microbiota, intestinal barrier, and bile acid signaling

The extremely low oral bioavailability of berberine has shifted attention from systemic exposure alone to the gastrointestinal tract as a major site of pharmacological action. Poor intestinal absorption, P-glycoprotein-mediated efflux, and extensive first-pass metabolism result in prolonged luminal exposure, allowing berberine to interact directly with intestinal microorganisms, epithelial and immune cells, bile acids, and nutrient-sensing pathways. The gut microbiota may also influence berberine pharmacokinetics. For example, microbial nitroreductases can convert berberine into the more readily absorbed dihydroberberine, which is subsequently reoxidized to berberine after entering intestinal tissues (Habtemariam, 2020). Thus, the gut microbiota functions not only as a pharmacological target but also as a metabolic interface that determines local and systemic drug exposure.

Berberine alters both the composition and metabolic activity of the gut microbiota. In experimental models of obesity, diabetes, hyperlipidemia, and fatty liver disease, it has been associated with enrichment of *Akkermansia*, *Bifidobacterium*, and selected short-chain fatty acid (SCFA)-producing taxa, including *Blautia*, *Allobaculum*, *Butyricicoccus*, and *Phascolarctobacterium*, together with reductions in several endotoxin-producing or metabolically unfavorable bacteria (Chen et al., 2026). These compositional changes may increase the production of acetate, propionate, and particularly butyrate. SCFAs provide energy to colonocytes, support mucus and epithelial homeostasis, and regulate metabolic and immune responses through mechanisms involving G-protein-coupled receptors, histone deacetylase inhibition, and intestinal hormone secretion. Increased butyrate availability has also been associated with enhanced GLP-1 and peptide YY release, improved insulin sensitivity, and reduced circulating glucose and lipid levels (Dong et al., 2022).

The intestinal barrier represents a second important level of regulation. Berberine has been reported to enhance the expression or organization of tight-junction and mucus-associated proteins, including ZO-1, occludin, claudins, and MUC2, thereby reducing intestinal permeability and microbial translocation (Cheng et al., 2022). Lower portal delivery of lipopolysaccharide may subsequently suppress TLR4/MyD88/NF-κB signaling and downstream production of TNF-α, IL-1β, IL-6, and other inflammatory mediators. By attenuating metabolic endotoxemia, berberine may interrupt the gut–adipose, gut–liver, and gut–immune inflammatory circuits that contribute to insulin resistance and hepatic injury. In selected models, enrichment of *Akkermansia muciniphila* has been linked to improved mucus-layer integrity and reduced bacterial translocation, although this response is not consistently observed across all disease settings (Zhang et al., 2020).

Bile acid metabolism provides a further mechanistic bridge between intestinal exposure and systemic metabolic regulation. Berberine can reduce the abundance or activity of bile salt hydrolase-producing bacteria, including members of *Clostridium* clusters XIVa and IV, thereby decreasing bile acid deconjugation and increasing luminal tauro-conjugated bile acids such as taurocholic acid (Cheng et al., 2022). The resulting activation of the ileal FXR–FGF15/19 axis can modify hepatic bile acid synthesis and lipid handling and may reduce the expression of hepatic lipid-uptake proteins such as CD36. Berberine-induced changes in primary and secondary bile acid pools may also influence TGR5-dependent glucose, energy, and inflammatory signaling, although direct evidence for TGR5 as a principal mediator remains less developed than that for intestinal FXR.

Together, these mitochondrial, metabolic, inflammatory, and gut-centered mechanisms define the multitarget pharmacological profile of BBR. However, several of these pathways are also modulated by other natural compounds, making it important to distinguish shared mechanisms from features that may be particularly characteristic of BBR.

### 4.6 Comparison with other natural modulators of metabolic inflammation

Berberine is not unique in targeting pathways involved in metabolic inflammation. Curcumin, resveratrol, and quercetin also modulate redox-sensitive inflammatory signaling, endogenous antioxidant defenses, mitochondrial function, and glucose–lipid homeostasis. However, the relative emphasis of these mechanisms differs among compounds. Curcumin is characterized predominantly by TLR4/MyD88/NF-κB and ROS–MAPK regulation, Nrf2-dependent antioxidant signaling, and AMPK-associated mitochondrial quality control. Resveratrol shows a prominent SIRT1/AMPK/PGC-1α signature linked to mitochondrial biogenesis, redox adaptation, and host–microbiota interactions, whereas quercetin is more strongly associated with flavonol-dependent ROS/NOX regulation, Nrf2 signaling, and kinase- and cytokine-related immunometabolic effects.

The distinguishing feature of BBR is therefore not an entirely unique molecular target, but the breadth with which several mechanistically connected regulatory layers converge within its pharmacology. Partial mitochondrial Complex I restraint and AMPK-centered energy sensing are linked to glucose–lipid reprogramming and inflammatory control, while prolonged intestinal exposure, microbial biotransformation, epithelial barrier regulation, and bile acid signaling provide an additional gut-centered layer of action. Circadian regulation may represent a further temporal dimension of this network, although this aspect remains considerably less established than its canonical metabolic and inflammatory mechanisms. Supplementary Table S1 summarizes these differences in mechanistic emphasis, translational evidence, and pharmacological limitations. Because direct head-to-head studies are scarce and formulations, doses, populations, and endpoints vary substantially, the comparison is intended to clarify pharmacological positioning rather than establish comparative therapeutic efficacy.

## 5 Circadian regulation as a temporal layer of berberine action in metabolic inflammation

### 5.1 Circadian regulation as a temporal layer of metabolic inflammatory networks

Circadian disruption acts as a temporal amplifier of metabolic inflammation by uncoupling nutrient flux, mitochondrial activity, intestinal function, bile acid metabolism, and immune responsiveness from daily behavioral rhythms. The relevance of circadian regulation to BBR arises because the major pathways described are themselves rhythmically organized. AMPK-associated energy sensing, mitochondrial substrate oxidation, inflammatory transcription, intestinal barrier function, bile acid signaling, and microbial metabolism fluctuate across the light–dark and feeding–fasting cycles.

Therefore, circadian regulation should not be viewed as an additional isolated mechanism parallel to mitochondrial, metabolic, inflammatory, and gut-mediated actions. Rather, it provides a timing framework that may determine when and where these mechanisms become most responsive to BBR. This perspective is particularly relevant for metabolic diseases, in which feeding time, sleep disruption, shift work, and social jetlag may aggravate insulin resistance, hepatic steatosis, adipose inflammation, intestinal permeability, and abnormal cytokine rhythms.

At the molecular level, circadian organization is maintained by transcriptional–translational feedback loops involving CLOCK, BMAL1, PER, CRY, REV-ERB, and ROR proteins. These clock components regulate metabolic genes directly and interact with nutrient-sensing and inflammatory pathways. The following sections therefore focus on two currently supported circadian interfaces of BBR: BMAL1/CLOCK-associated regulation of hepatic metabolic homeostasis and REV-ERBα-associated regulation of time-dependent inflammatory responsiveness.

### 5.2 BMAL1/CLOCK-dependent metabolic regulation by berberine

Against this background, the hepatic BMAL1/CLOCK axis provides one of the most direct mechanistic links between berberine, peripheral clock function, and metabolic homeostasis. Evidence from hepatic metabolic models suggests that this transcriptional complex may contribute functionally to the glucose-, lipid-, and redox-regulatory effects of berberine (Ye et al., 2023). In high-fat diet-induced fatty liver and insulin-resistant hepatocyte models, metabolic and redox disturbances were accompanied by reduced CLOCK and BMAL1 expression. Berberine improved glucose utilization, lipid accumulation, insulin responsiveness, and oxidative stress while partially restoring CLOCK and BMAL1 expression (Xu Z. H. et al., 2025).

Time-course analyses in insulin-resistant hepatocytes further suggested that metabolic stress reduced the amplitude and altered the phase of CLOCK and BMAL1 expression, whereas berberine partially normalized these patterns. More importantly, Bmal1 silencing attenuated the effects of berberine on glucose uptake and reactive oxygen species accumulation, supporting a functional contribution of BMAL1 rather than a purely correlative association (Wang Y. et al., 2024). These observations provide a mechanistic entry point linking berberine-mediated metabolic regulation with the peripheral clock. They suggest that berberine may improve hepatic metabolic homeostasis partly by restoring coordination among clock-controlled transcription, insulin responsiveness, and redox balance.

### 5.3 REV-ERBα and circadian pharmacodynamics

REV-ERBα represents a second interface between the circadian clock, metabolism, and inflammation. As a transcriptional repressor in the secondary clock loop, it regulates lipid metabolism and innate immune signaling, including repression of NLRP3-associated inflammatory transcription.

In a murine model of chronic colitis, the severity of inflammation and the expression of inflammatory mediators varied across the day. Berberine produced stronger anti-inflammatory activity at ZT10 than at ZT2 despite comparable systemic exposure at the two dosing times. Its activity was attenuated in REV-ERBα-deficient cells, whereas reporter experiments suggested that berberine enhanced REV-ERBα-mediated repression of Bmal1 and Nlrp3 (Wu et al., 2020; Zhou et al., 2020). These findings provide proof-of-concept that the pharmacodynamic response to berberine may depend on circadian phase.

The apparently divergent effects of berberine on BMAL1 in hepatic and colonic models should not necessarily be regarded as contradictory. In metabolically impaired hepatocytes, berberine restored pathologically reduced BMAL1 expression and oscillation, whereas in inflamed colonic tissue it enhanced REV-ERBα-dependent repression of Bmal1 and inflammatory outputs (Zhou et al., 2020). These differences may reflect tissue type, disease state, phase, and the regulatory level being measured.

The colitis study supports time-dependent anti-inflammatory pharmacology but does not establish an optimal dosing window for obesity, diabetes, or MASLD. Evidence for circadian pharmacodynamics is currently stronger in intestinal inflammation than in metabolic disease models. Direct metabolic-disease studies are therefore required before chronotherapeutic recommendations can be made.

### 5.4 Chronometabolic implications and future directions

Current evidence supports BBR as a clock-interacting metabolic modulator. In hepatic metabolic models, BBR partially restores BMAL1/CLOCK expression and rhythmicity in association with improved glucose utilization, lipid handling, insulin responsiveness, and redox balance. In inflammatory intestinal models, BBR enhances REV-ERBα-dependent repression of Bmal1 and Nlrp3 and shows dosing-time-dependent anti-inflammatory efficacy. Together, these findings suggest that circadian regulation may influence both the metabolic and anti-inflammatory actions of BBR.

**FIGURE 2:**
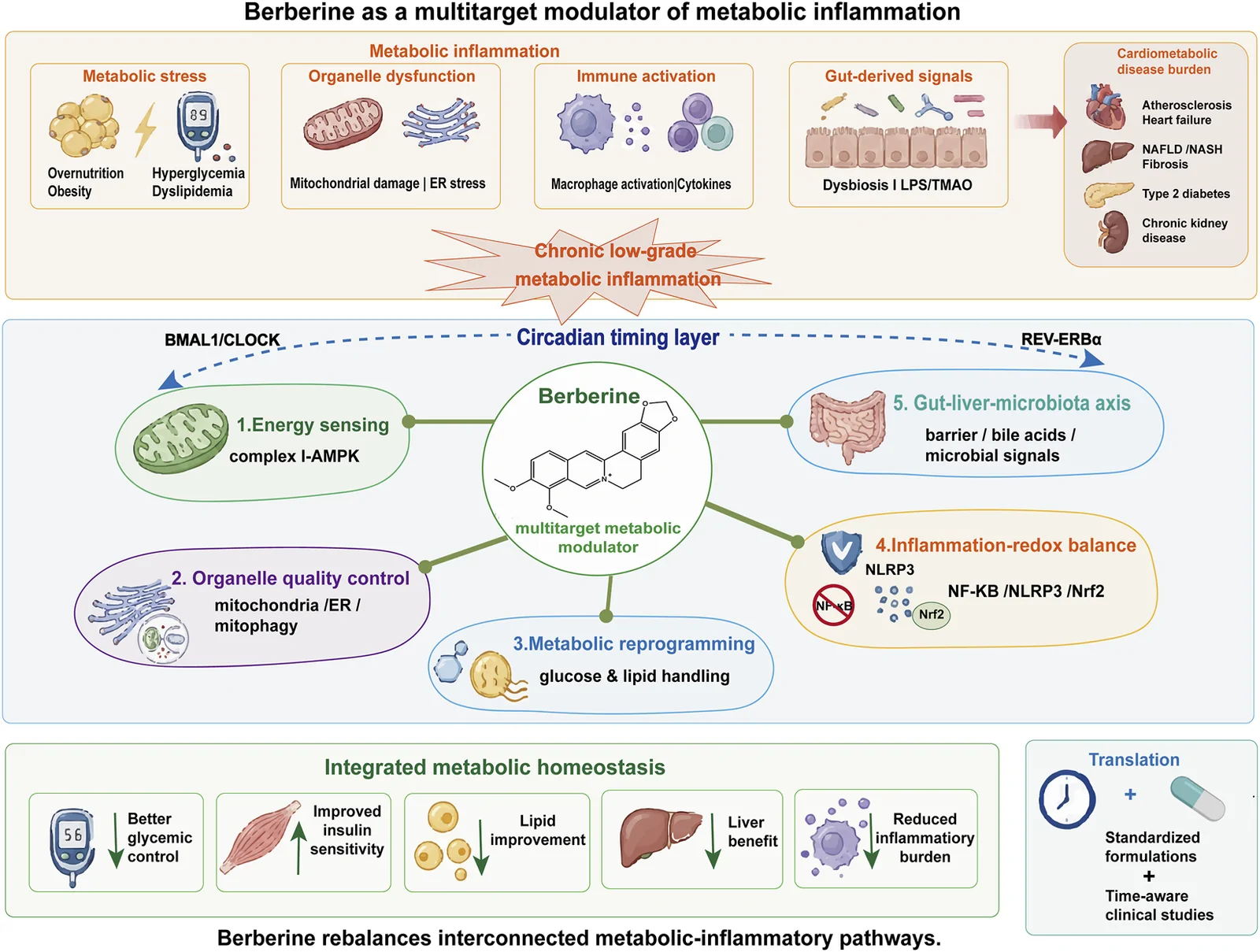
Circadian regulation as an emerging dimension of berberine action in metabolic inflammation. Circadian disruption caused by irregular light exposure, sleep restriction, shift work, mistimed feeding, and psychological stress may aggravate insulin resistance, hepatic steatosis, adipose inflammation, gut dysrhythmia, mitochondrial redox imbalance, and abnormal inflammatory rhythms. Berberine may interact with the CLOCK/BMAL1–PER/CRY–REV-ERB/ROR network and its cross-talk with AMPK/SIRT1, MAPK/NF-κB, NLRP3, mitochondrial redox regulation, and gut microbial rhythmicity.

However, the chronometabolic field remains at an early stage. Most studies of BBR have measured metabolic, inflammatory, microbiota, or bile acid endpoints at a single time point, making it difficult to determine whether BBR restores rhythmicity or simply changes pathway activity. But circadian biology does not replace the multitarget mechanism of BBR; it refines it by adding temporal precision. BBR may be most effective when its intestinal, hepatic, mitochondrial, and inflammatory actions are aligned with endogenous metabolic rhythms (Figure 2).

## 6 Therapeutic potential of berberine in metabolic diseases

### 6.1 Obesity and metabolic syndrome

Obesity is a disorder of adipose-tissue expandability, endocrine function, immune homeostasis, and systemic substrate distribution. When adipose storage capacity is exceeded, adipocyte hypertrophy, hypoxia, fibrosis, altered adipokine secretion, and immune-cell recruitment promote insulin resistance and ectopic lipid deposition. Metabolic syndrome represents the clinical convergence of these abnormalities with dysglycemia, dyslipidemia, hypertension, and increased cardiovascular risk.

Preclinical evidence indicates that berberine may improve adipose tissue function by reducing adipogenesis and fibrotic remodeling, enhancing lipid oxidation and thermogenic programming, and attenuating inflammatory activation (Bandala et al., 2024; Zhang et al., 2025). Intestinal and microbial effects may further influence energy harvest, enteroendocrine signaling, and metabolic endotoxemia. Beyond regulating adipogenesis and energy metabolism, berberine may reduce obesity by limiting intestinal lipid absorption. In diet-induced obese mice, berberine decreased body weight without suppressing food intake and improved glucose and lipid homeostasis. Mechanistically, it promoted the zippering of intestinal lacteal endothelial junctions through inhibition of the RhoA/ROCK signaling pathway, thereby restricting dietary lipid uptake (Wang H. et al., 2024). Consistent effects on junction maturation were observed in human lymphatic endothelial cells, suggesting that remodeling of the intestinal lacteal barrier represents an additional gut-localized mechanism underlying the anti-obesity activity of berberine.

However, the translation of these mechanisms into clinically meaningful weight reduction remains limited. Human studies more consistently suggest improvements in waist-related indices, body composition, adipokines, insulin sensitivity, and circulating lipids than substantial or sustained weight loss (Kong et al., 2025). Berberine may therefore have greater value as an adjunct for obesity-associated metabolic dysfunction than as a primary anti-obesity treatment.

### 6.2 Type 2 diabetes mellitus and diabetic complications

Type 2 diabetes mellitus arises from the interaction of insulin resistance, excessive hepatic glucose production, impaired peripheral glucose utilization, inadequate β-cell compensation, and chronic inflammatory stress. Berberine addresses several of these pathological components simultaneously through systemic, hepatic, muscular, intestinal, and gut microbial actions (Shrivastava et al., 2023).

In T2DM, berberine improves glucose homeostasis by enhancing insulin sensitivity and glucose uptake, promoting glycogen synthesis, and suppressing hepatic gluconeogenesis. These effects are associated with the regulation of AMPK, AKT, and glucose transporter signaling, together with attenuation of oxidative stress and metabolic inflammation (Liu et al., 2025). Clinical studies have reported reductions in fasting and postprandial glucose, glycated hemoglobin, insulin resistance, and triglycerides. Some small trials have observed effects approaching those of metformin, but their limited size, short duration, and methodological heterogeneity do not establish therapeutic equivalence (Bhushan et al., 2026). The most defensible clinical positioning is as an adjunct in insulin-resistant T2DM, especially when dyslipidemia or intestinal metabolic dysfunction coexist.

Preclinical studies also suggest protective effects against diabetic nephropathy, neuropathy, endothelial dysfunction, cardiomyopathy, and other complications through regulation of mitochondrial homeostasis, autophagy, oxidative stress, and fibrosis. Human evidence for preventing or delaying these complications remains substantially weaker than evidence for short-term glucose lowering.

### 6.3 MASLD/MASH and hepatic metabolic inflammation

Metabolic dysfunction-associated steatotic liver disease (MASLD) represents the hepatic manifestation of systemic metabolic dysfunction. Hepatic steatosis develops when fatty acid delivery and *de novo* lipogenesis exceed mitochondrial oxidation and lipid export, whereas progression to metabolic dysfunction-associated steatohepatitis (MASH) and fibrosis is driven by lipotoxic stress, inflammation, and hepatic stellate-cell activation (Ren et al., 2026).

Berberine may intervene at multiple stages of this disease continuum. By improving systemic insulin sensitivity, it can reduce hepatic fatty acid influx and lipid synthesis while enhancing fatty acid utilization. Within hepatocytes, berberine suppresses SREBP-1c- and acetyl-CoA carboxylase-associated lipogenesis and promotes PPARα- and CPT-1-mediated fatty acid oxidation. It may also directly bind histone deacetylase 1 and inhibit the NF-κB/HDAC1/SREBP-1c axis, thereby linking the suppression of inflammatory signaling with reduced abnormal lipid synthesis (Fan et al., 2026).

The gut–liver axis provides an additional mechanism, particularly given the high intestinal exposure of orally administered berberine. In methionine- and choline-deficient diet-induced MASH mice, berberine alleviated hepatic steatosis and inflammation, increased intestinal Claudin-1 and MUC2 expression, limited bacterial translocation, and increased the abundance of Akkermansia muciniphila (Ren et al., 2026). Antibiotic depletion weakened these benefits, whereas fecal microbiota transplantation from berberine-treated donors partially reproduced them, supporting a functional role for microbiota remodeling. Berberine may further inhibit microbial bile salt hydrolase, reshape the conjugated bile acid pool, and activate ileal FXR–FGF15 signaling (Xu J. et al., 2025). A meta-analysis of 10 randomized controlled trials involving 811 patients diagnosed with NAFLD under the previous nomenclature found that berberine improved liver enzymes, circulating lipids, insulin resistance, and body mass index (Nie et al., 2024). These findings provide supportive, although indirect, clinical evidence for berberine in MASLD.

Translationally, berberine ursodeoxycholate (HTD1801) produced a higher magnetic resonance imaging response rate than placebo in an 18-week phase 2 study of patients with presumed MASH and type 2 diabetes (Wong et al., 2025). Overall, berberine is best regarded as a multitarget modulator of insulin resistance, hepatic steatosis, intestinal barrier dysfunction, and metabolic inflammation. More broadly, nanocarrier systems are being developed to improve stability, targeting, and antifibrotic delivery of plant-derived isolates in liver disease (Kashyap, 2025), providing a formulation rationale that may be relevant to future liver-directed BBR delivery, although direct evidence remains necessary.

### 6.4 Dyslipidemia, atherosclerosis, and cardiovascular metabolic risk

Dyslipidemia and vascular inflammation connect metabolic dysfunction to atherosclerotic cardiovascular disease. Berberine can influence hepatic cholesterol handling, LDL receptor-related pathways, intestinal cholesterol absorption, bile acid metabolism, triglyceride turnover, and systemic glucose–lipid coordination.

Preclinical studies additionally suggest favorable effects on endothelial function, oxidative stress, adhesion molecules, foam-cell formation, and plaque-associated inflammation. These vascular mechanisms are biologically plausible, but most have not been validated using clinical cardiovascular outcomes.

Berberine exerts lipid-lowering effects through complementary hepatic and intestinal mechanisms. It increases hepatic low-density lipoprotein receptor expression and has been reported to suppress PCSK9 expression, thereby promoting the clearance of circulating LDL cholesterol (Coppinger et al., 2024). Berberine may also reduce intestinal cholesterol absorption and facilitate hepatic cholesterol conversion and biliary excretion. These coordinated actions contribute to reductions in LDL cholesterol and may partly underlie its potential cardiovascular benefits (Nie et al., 2024). However, evidence directly linking berberine treatment to reductions in major cardiovascular events remains limited. Clinical evidence generally supports modest reductions in total cholesterol, LDL cholesterol, and triglycerides. Interpretation is complicated by the frequent use of multicomponent nutraceuticals containing red yeast rice, policosanols, phytosterols, or other active substances. Improvements observed with these products cannot be attributed exclusively to berberine.

Evidence that berberine prevents myocardial infarction, stroke, revascularization, or cardiovascular death is lacking. It should therefore not replace statins or other guideline-directed therapies. Its potential adjunctive role in mixed dyslipidemia, statin intolerance, or combined glucose–lipid abnormalities requires prospective evaluation with clearly characterized parent berberine preparations.

### 6.5 Polycystic ovary syndrome and endocrine–metabolic disorders

Polycystic ovary syndrome is characterized by a reciprocal relationship among insulin resistance, compensatory hyperinsulinemia, adipose dysfunction, dyslipidemia, and androgen excess. Berberine may improve this phenotype predominantly through systemic insulin sensitization and metabolic regulation rather than through a direct ovarian action alone.

Small clinical studies have reported improvements in insulin resistance, lipid profiles, waist-related measurements, sex hormone-binding globulin, and selected reproductive parameters. However, diagnostic heterogeneity, background hormonal treatment, fertility protocols, and inconsistent endpoint selection limit the strength of these conclusions.

Metabolic and reproductive outcomes should be distinguished. Evidence supporting improved metabolic status does not necessarily establish effects on ovulation, pregnancy, miscarriage, or live birth. Berberine cannot currently be considered a primary fertility treatment, and reproductive, pregnancy, neonatal, and long-term offspring safety require dedicated investigation.

### 6.6 Cross-disease interpretation

The strength of evidence differs substantially among metabolic indications (Suren Garg et al., 2023). Clinical support is relatively strongest for glycemic and lipid-related surrogate outcomes, while findings for hepatic steatosis are promising but heterogeneous. Evidence remains weaker for substantial weight loss, fibrosis regression, diabetic complications, cardiovascular events, and definitive reproductive outcomes.

This discrepancy indicates that broad preclinical polypharmacology has not translated uniformly into clinical benefit (Lu et al., 2022). Berberine is most likely to be useful in patients with overlapping insulin resistance, dyslipidemia, hepatic fat accumulation, low-grade inflammation, or intestinal metabolic dysfunction. Future development should focus on metabolically defined subgroups rather than positioning berberine as a universal therapy for all metabolic disorders.

## 7 Discussion

### 7.1 Interpretation and quality of the clinical evidence

Clinical evidence supports the therapeutic potential of BBR across several metabolic indications, particularly for intermediate outcomes related to glycemic control, insulin resistance, circulating lipids, body weight-related indices, and hepatic steatosis. These outcomes are biologically consistent with the multitarget mechanisms discussed above and support the positioning of BBR as an adjunctive metabolic modulator rather than a replacement for established disease-specific therapies.

The most consistent clinical signals have been observed in glucose and lipid metabolism. Studies in patients with type 2 diabetes or dysglycemia have reported improvements in fasting glucose, HbA1c, insulin resistance, and lipid parameters, while studies in dyslipidemia have generally shown reductions in total cholesterol, LDL cholesterol, and triglycerides. In steatotic liver disease, available trials and pooled analyses suggest improvements in aminotransferases, insulin resistance, circulating lipids, body mass index, and imaging-related hepatic fat indices. These findings indicate that BBR acts most plausibly on the shared metabolic-inflammatory substrate linking T2DM, dyslipidemia, obesity, and MASLD/MASH (Supplementary Table S1).

The interpretation of these data requires careful product distinction. Parent BBR or berberine hydrochloride, botanical extracts, enhanced-bioavailability formulations, probiotic combinations, multicomponent nutraceuticals, and structural derivatives such as berberine ursodeoxycholate differ in pharmacokinetics, tissue exposure, mechanisms, and safety. Results from combination products or derivatives should therefore be used to support berberine-based therapeutic strategies but should not be attributed uncritically to conventional BBR monotherapy.

There is no universally approved standard therapeutic dose of BBR for metabolic disease because product composition and regulatory status differ across jurisdictions. In conventional oral BBR/berberine hydrochloride trials, however, 0.5 g two or three times daily (approximately 1.0–1.5 g/day) is a commonly investigated regimen, with treatment generally lasting several weeks to a few months depending on the indication and study design (Xu et al., 2021). For example, Yin et al. (2008) administered BBR at 0.5 g three times daily for 3 months in patients with type 2 diabetes, and the early hypercholesterolemia study by Kong et al. (2004) also used a 3-month treatment course; by contrast, the phase 2 study of berberine ursodeoxycholate (HTD1801) in patients with MASH and type 2 diabetes lasted 18 weeks (Wong et al., 2025). These regimens should be interpreted as study-specific exposures rather than a universally established dose, and dose equivalence cannot be assumed across conventional BBR, combination products, structural derivatives, or enhanced-bioavailability formulations.

The current evidence base is strongest for metabolic surrogate endpoints and weaker for long-term disease modification. Improvements in HbA1c, LDL cholesterol, liver enzymes, or hepatic fat are clinically meaningful signals, but they do not by themselves establish reductions in cardiovascular events, advanced fibrosis, organ failure, reproductive outcomes, or mortality. Accordingly, the next stage of clinical translation should not ask whether BBR is a universal substitute for metformin, statins, anti-obesity agents, or antifibrotic therapies. Instead, it should determine which standardized BBR preparation, at which dose and timing, benefits which metabolic phenotype, and through which measurable biological pathway (Xu et al., 2021).

Several limitations should temper interpretation of the current evidence. First, mechanistic evidence remains dominated by cell and animal studies, often using concentrations or exposure patterns that may not be reproduced in human target tissues. Second, many clinical trials are small, short, and heterogeneous with respect to diagnosis, background therapy, product identity, dose, and endpoints, and most rely on surrogate metabolic outcomes rather than major cardiovascular, hepatic, renal, or reproductive events. Third, pooling conventional BBR with botanical extracts, multicomponent nutraceuticals, probiotic combinations, or structural derivatives may obscure formulation-specific efficacy and safety. Fourth, microbiome and circadian studies frequently rely on association-based readouts or limited sampling times, while direct target engagement, exposure-response relationships, and long-term pharmacovigilance remain incompletely characterized. Publication and selective-reporting bias may further influence the apparent consistency of the literature. These limitations preclude strong claims of disease modification, optimal dosing, or comparative efficacy against established therapies.

### 7.2 Pharmacokinetic, formulation, and safety barriers

The clinical translation of berberine is constrained by its pharmacokinetic–pharmacodynamic paradox. Low and variable plasma exposure contrasts with its broad biological activity, suggesting that intestinal retention, microbial biotransformation, enterohepatic circulation, active metabolites, and local hepatic or intestinal effects may contribute substantially to efficacy. Plasma parent-drug concentration alone may therefore be an inadequate indicator of target engagement. Formulation heterogeneity further complicates interpretation. Clinical and commercial products vary in botanical source, purity, salt form, particle size, dissolution behavior, dose, dosing frequency, and active-metabolite exposure. Strategies designed to increase absorption may alter the relative contribution of intestinal and systemic actions. From a clinical-development perspective, an exposure-enhancing formulation would be most valuable if it reduces dose burden or pharmacokinetic variability, improves access to the intended extraintestinal target, or improves efficacy/tolerability in a measurable manner. Enhanced bioavailability should not automatically be assumed to improve therapeutic value. The optimal formulation may differ depending on whether the intended target is located primarily in the intestine, liver, vascular system, or other peripheral tissues.

Commercially available BBR products should be distinguished from novel delivery systems. Conventional commercial products are most commonly supplied as immediate-release berberine hydrochloride or other salts in tablets/capsules, botanical extracts, or multicomponent nutraceuticals. They have lower manufacturing complexity and substantially more human-use experience, but retain the characteristic low and variable oral absorption of BBR and may differ in purity, dissolution behavior, labeled dose, and co-ingredients. In contrast, novel formulations—including lipid/polymer nanoparticles, liposomes or micelles, phospholipid complexes, cocrystals and engineered salts, carrier-free nanoassemblies, and local sustained-release systems—are designed to improve solubility and permeability, exploit intestinal or lymphatic transport, prolong release, or direct exposure to selected tissues (Cui et al., 2024; Ming et al., 2024; Xu Y. et al., 2024; Surampudi et al., 2025; Thool and Pokharkar, 2025). The formulation studies summarized in Section 2.2 demonstrate marked increases in systemic or local exposure, but most remain preclinical or early translational. Improved pharmaceutical performance should therefore not be equated with established clinical superiority; scale-up, batch reproducibility, excipient safety, stability, bioequivalence, cost, and formulation-specific regulatory characterization remain important barriers.

Berberine is generally well tolerated during short-term administration, with constipation, diarrhea, nausea, and abdominal discomfort being the most commonly reported adverse effects. However, adverse-event collection has not been standardized, and long-term safety remains insufficiently characterized. Potential interactions involving intestinal transporters and drug-metabolizing enzymes require systematic evaluation, particularly in patients receiving glucose-lowering drugs, statins, antihypertensive agents, antiplatelet or anticoagulant therapies, immunomodulators, or drugs with narrow therapeutic indices. Special attention is also required in older adults, patients with hepatic or renal dysfunction, and individuals receiving multiple medications. Pregnancy, lactation, fertility, and neonatal safety remain inadequately defined. This limitation is particularly relevant to the proposed use of berberine in PCOS. The consequences of sustained alteration of the intestinal microbiota and bile acid pool also require long-term investigation.

Clinically documented drug-interaction data remain limited but are relevant. In healthy volunteers, BBR 300 mg three times daily for 2 weeks reduced CYP2D6, CYP2C9, and CYP3A4 activities and increased midazolam exposure (Guo et al., 2012). In renal-transplant recipients, coadministration of BBR 0.2 g three times daily increased cyclosporine blood exposure (Wu et al., 2005), indicating a clinically important interaction potentially involving CYP3A4- and transporter-dependent disposition. These findings support caution and therapeutic monitoring when BBR is combined with narrow-therapeutic-index drugs or clinically sensitive CYP/transporter substrates. Pharmacodynamic additivity, such as greater glucose- or blood-pressure lowering when BBR is combined with antidiabetic or antihypertensive therapy, should also be considered even when a specific pharmacokinetic interaction has not been demonstrated.

Safety and regulatory interpretation should also be formulation- and jurisdiction-specific. Short-term studies generally identify gastrointestinal symptoms as the most frequent adverse events, but systematic long-term safety data and pharmacovigilance remain limited. Particular concerns include potential hypoglycemia or hypotension in susceptible patients, clinically relevant enzyme/transporter-mediated interactions, and insufficient evidence for pregnancy, lactation, neonatal exposure, and long-term reproductive safety. Regulatory classification differs internationally. In Europe, the European Food Safety Authority has undertaken a formal safety-assessment process for berberine-containing plant preparations. Novel high-bioavailability or nano-enabled formulations may require additional evaluation because excipient composition, particle characteristics, altered systemic exposure, and tissue distribution can modify risk. Standardized identity and purity, GMP-quality manufacture, defined exposure specifications, interaction studies, long-term adverse-event surveillance, and formulation-specific regulatory assessment are therefore prerequisites for wider clinical translation.

### 7.3 Priorities for mechanistic and clinical research

Future research should move from broad empirical evaluation toward mechanism-guided clinical development. The most promising direction is not to test BBR as a nonspecific supplement for all metabolic disorders, but to identify responder phenotypes characterized by overlapping insulin resistance, dyslipidemia, hepatic fat accumulation, gut barrier dysfunction, bile acid dysregulation, or low-grade inflammatory activation. In such populations, BBR has a strong mechanistic rationale because its intestinal, hepatic, mitochondrial, and inflammatory actions converge on shared metabolic-inflammatory pathways. A translational framework for BBR should include five elements: standardized product identity, exposure–response characterization of parent compound and active metabolites, causal target validation, disease-specific mechanistic biomarkers, and clinically meaningful endpoints. Chronometabolic trial design may further refine this framework by incorporating feeding time, sleep schedule, dosing time, and rhythm-related biomarkers. This strategy would transform BBR development from general multitarget pharmacology into precision metabolic intervention.

The mechanistic literature also differs substantially in the strength of experimental validation. Relatively strong causal support exists for mitochondrial complex I-linked energy stress followed by AMPK activation (Yu et al., 2021), BMAL1 involvement supported by gene-silencing experiments in hepatic models (Wang H. et al., 2024), and REV-ERBα-dependent time-of-day anti-inflammatory pharmacodynamics supported by loss-of-function and reporter experiments (Zhou et al., 2020). Experimental work also supports gut-centered mechanisms such as microbial biotransformation, barrier regulation, and bile-acid signaling, although their relative contribution differs among disease models. By contrast, several frequently cited mechanisms should remain working models rather than direct-target claims: NLRP3 suppression is reproducible, but direct molecular binding of BBR to NLRP3 is not established; microbiota taxonomic shifts are not uniformly causal across diseases; adipose browning remains predominantly preclinical; and the concept of BBR as a systemic “multitarget recalibrator” is an integrative framework rather than a single molecular mechanism proven by one experiment. Future studies should prioritize genetic rescue or loss-of-function approaches, direct binding and target-engagement assays, exposure-matched models, and human mechanistic biomarkers to distinguish causal targets from downstream associations.

## 8 Conclusion

Berberine is a plant-derived isoquinoline alkaloid whose metabolic effects are best understood through network pharmacology rather than a single-target model. Its actions appear to originate from intestinal and mitochondrial interfaces and propagate through cellular energy sensing, metabolic adaptation, organelle quality control, inflammatory and redox regulation, and inter-organ communication (Cicero and Baggioni, 2016; Qin et al., 2024). This network is highly context-dependent. The direction and magnitude of berberine activity vary with dose, tissue, metabolic state, microbial environment, formulation, and duration of exposure. Alteration of a signaling pathway should therefore not automatically be interpreted as direct target engagement or universal therapeutic activity.

Circadian regulation adds a temporal dimension to this framework. Preliminary evidence supports interactions with BMAL1/CLOCK-associated metabolic regulation and REV-ERBα-dependent inflammatory responsiveness, but direct evidence for restoration of systemic rhythmicity and clinically meaningful time-of-day effects remains limited. Clinical studies support modest benefits for glycemic control, insulin resistance, dyslipidemia, and selected features of steatotic liver disease, whereas evidence for long-term organ protection and major clinical outcomes remains insufficient. Berberine should therefore be positioned as a promising adjunctive metabolic modulator rather than a substitute for evidence-based pharmacotherapy.

The central translational question is no longer whether berberine affects multiple pathways, but which combinations of formulation, dose, tissue exposure, metabolic phenotype, microbiome state, and administration time generate clinically meaningful network regulation. Addressing this question will determine whether berberine can progress from a broadly used natural compound to a standardized component of precision metabolic and chronometabolic therapy.
