# Supplementary material for: Berberine as a multitarget modulator of metabolic inflammation: molecular mechanisms, circadian regulation, and translational perspectives
Source: Front Pharmacol. 2026 Sep 8;17:1935151. doi: 10.3389/fphar.2026.1935151 (PMC13594412; doi:10.3389/fphar.2026.1935151)
Supplement: Supplementary file 1 [file Table1.docx]

Supplementary Table S1. Registered clinical trials of berberine and berberine-based interventions in metabolic diseases

| Disease/condition | Status | Number of patients | Intervention measures | Duration / follow-up | Completion date | Identifier | Study title | Notes |
| --- | --- | --- | --- | --- | --- | --- | --- | --- |
| Metabolic Syndrome | Completed | 66 | Armolipid Plus | 18 weeks | 2010-07 | NCT01087632 | Armolipid Plus and Metabolic Syndrome | * |
| Metabolic Syndrome | Completed | 150 | Armolipid Prev | 12 months | 2014-10 | NCT01293162 | The Role of a Combination of Nutraceuticals in the Control of Cardiovascular Risk | * |
| Metabolic Syndrome | Completed | 28 | CAR-191; Placebo | 12 weeks | 2013-04 | NCT01696448 | The BANGALORE Study; Combination of Berberine, Lipoic Acid, and Picrorhiza | * |
| Metabolic Syndrome\|Insulin-resistance\|Left Ventricular Hypertrophy | Completed | 159 | Armolipid Plus; Placebo | 6 months | 2015-07 | NCT02295176 | Metabolic and Cardiovascular Effects of Armolipid Plus in Subjects With Metabolic Syndrome | * |
| Schizophrenia\|Schizophrenia Spectrum and Other Psychotic Disorders\|Metabolic Syndrome x | Completed | 113 | Berberine; Placebos; Antipsychotic Agents | 3 week | 2021-01-04 | NCT02983188 | Berberine as Adjuvant Treatment for Schizophrenia Patients | / |
| Metabolic Syndrome\|Female\|Schizophrenia | Completed | 36 | berberine | 8 weeks | 2017-12-30 | NCT03251716 | Berberine Treat Metabolic Syndrome in Schizophrenia | / |
| Schizophrenia\|Metabolic Syndrome\|Berberine\|Metformin | Unknown | 100 | Berberine; Metformin | 12 weeks | 2023-12-01 | NCT03708549 | Comparison of Berberine and Metformin for the Treatment for MS in Schizophrenia Patients | * |
| Metabolic Syndrome | Completed | 15 | Berberine; Identical Placebo | Not reported in uploaded file | 2025-04-01 | NCT03976336 | Evaluating the Tolerability and Effects of Berberine on Major Metabolic Biomarkers: A Pilot Study | / |
| Metabolic Syndrome\|HIV-1-infection\|Glucose Intolerance\|Dyslipidemias\|Lipodystrophy\|Lipoatrophy | Unknown | 40 | Berberine; Placebo | 6 months | 2022-07 | NCT04860063 | Effect of Berberine on Metabolic Syndrome, Efficacy and Safety in Combination With Antiretroviral Therapy in PLWH. | * |
| Obesity\|Overweight\|Berberine | Completed | 120 | Berberine Hydrochloride group; nutritional plan; controlled moderate aerobic exercise | 3 months | 2020-02-15 | NCT06703086 | Gene Modulation of NLRP3, IL-1β and TNF-α in Peripheral Blood of Patients With Exogenous Obesity Treated With Berberine | / |
| HELICOBACTER PYLORI INFECTIONS\|Metabolic Syndrome | Recruiting | 120 | Vonoprazan-Amoxicillin Dual Therapy; Vonoprazan-Amoxicillin with Bifidobacterium tetravaccae; Vonoprazan-Amoxicillin with Berberine | 1 month | 2026-06-30 | NCT07306988 | Eradication of Helicobacter Pylori Improves Metabolic Syndrome Through Modulation of Gut Microbiota. | / |
| Cardiometabolic Syndrome | Not Yet Recruiting | 40 | Berberine; Placebo | Not reported in uploaded file | 2029-01-01 | NCT07541898 | Effects of Berberine on Cardiometabolic Outcomes | / |
| Perimenopausal Women\|Body Composition Changes | Not Yet Recruiting | 80 | Nutritional Supplement; Placebo | 12 weeks | 2026-12 | NCT07615777 | Nutritional Supplementation on Physiological and Metabolic Markers in Females | / |
| Type 2 Diabetes | Completed | 70 | Berberine; Metformin | 13 weeks | 2004-12 | NCT00425009 | Therapeutic Effects of Berberine in Patients With Type 2 Diabetes | * |
| Diabetes Mellitus, Type 2\|Metabolic Syndrome | Completed | 120 | Berberine | Not reported in uploaded file | 2006-09 | NCT00462046 | Efficacy and Safety of Berberine in the Treatment of Diabetes With Dyslipidemia | / |
| Prediabetes | Withdrawn | 0 | Bifidobacterium viable pharmaceutics; Berberine Hydrochloride | 12 weeks | 2018-04 | NCT02082756 | Effects of Berberine Hydrochloride and Bifidobacterium in Prediabetes Prevention and Treatment | / |
| Type 2 Diabetes Mellitus | Withdrawn | 0 | Bifidobacterium viable pharmaceutics; Berberine Hydrochloride | 12 weeks | 2018-04 | NCT02084004 | Effects of Berberine Hydrochloride and Bifidobacterium in Diabetes Mellitus Prevention and Treatment | / |
| Hyperglycemia | Unknown | 200 | Berberine; Insulin; Insulin | 10 days | 2017-07 | NCT02806999 | The Therapeutic Effects of Insulin and Berberine on Stress Hyperglycemia | / |
| Diabetes Mellitus\|Chronic Kidney Disease | Completed | 800 | Berberine | 30 days | 2021-12 | NCT02808351 | Berberine Prevent Contrast-induced Nephropathy in Patients With Diabetes | / |
| Pre Diabetes | Completed | 126 | BGG; Placebo | Not reported in uploaded file | 2016-07 | NCT02834078 | Effect of BGG on Glucose Metabolism and Other Markers of Metabolic Syndrome | / |
| Type 2 Diabetes | Unknown | 400 | 1. Berberine hydrochloride tablets ; 2. ProMetS probiotics powder; 1. Berberine placebo tablets ; 2. ProMetS probiotics powder; 1. Berberine hydrochloride tablets; 2. Probiotics placebo powder; 1. Berberine placebo tablets; 2. Probiotics placebo powder | 13 weeks | 2019-05 | NCT02861261 | A Study on the Efficacy and Gut Microbiota of Berberine and Probiotics in Patients With Newly Diagnosed Type 2 Diabetes | * |
| Prediabetes\|Impaired Fasting Glucose\|Impaired Glucose Tolerance | Unknown | 28 | Berberine; Metformin | Not reported in uploaded file | 2023-12 | NCT03029390 | Effect of Berberine Versus Metformin on Glycemic Control, Insulin Sensitivity and Insulin Secretion in Prediabetes | * |
| Diabetes Mellitus | Completed | 15 | Berberine Chloride; Placebo | 2 weeks | 2020-02-17 | NCT03972215 | Berberine Hyperglycemic Clamp | / |
| Dysglycemia | Completed | 148 | Reglicem; Placebo | 3 months | 2019-08-31 | NCT04107987 | Berberine, Curcumin, Inositol, Banaba and Chromium Picolinate in Patients With Fasting Dysglycemia | * |
| Type 1 Diabetes Mellitus\|Autoimmune Diabetes | Not Yet Recruiting | 240 | Berberine; Inulin; Berberine placebo tablets; Inulin placebo tablets | Not reported in uploaded file | 2026-12 | NCT04698330 | Effects Of Berberine Plus Inulin On Diabetes Care in Patients With LADA | * |
| Glycemia | Completed | 5 | Berberine; Dihydroberberine 200; Dihydroberberine 100; Placebo | 30 days | 2020-11-04 | NCT05021341 | Bioavailability of Berberine and Dihydroberberine and Their Impact on Glycemia | / |
| Glucose Metabolism Disorders | Unknown | 50 | Berberine Phytosome | 4 weeks | 2022-07-25 | NCT05031715 | Berberine and Altered Fasting Glucose | / |
| Metabolic Syndrome | Not Yet Recruiting | 5200 | Berberine; Healthy lifestyle intervention | Not reported in uploaded file | 2028-12 | NCT05105321 | Cardiovascular Diseases and Diabetes Prevention Programme in Metabolic Syndrome (CDPP) | / |
| Diabetes | Completed | 44 | Berberine+Cinnamon (TDS); Placebo (TDS) | Not reported in uploaded file | 2023-02-10 | NCT05570357 | Berberine and Cinnamon in Management of Diabetes | * |
| Prediabetes | Completed | 110 | Akkermansia muciniphila; Berberine; Placebo | 12weeks | 2025-06-30 | NCT05720299 | A Clinical Study to Evaluate the Effects of Akkermansia Muciniphila and Berberine on Prediabetes Among Obese Subjects. | * |
| PreDiabetes\|Abdominal Obesity\|Hypertension\|Dyslipidemias\|Atherosclerotic Cardiovascular Disease | Active Not Recruiting | 2024 | Berberine plus lifestyle intervention; Placebo plus lifestyle intervention | Not reported in uploaded file | 2026-12-31 | NCT05749874 | Effects of Berberine on Preventing Cardiovascular Disease and Diabetes Mellitus | * |
| Diabetes Mellitus | Completed | 16 | Berberine Chloride; Placebo | Not reported in uploaded file | 2023-04-20 | NCT05947370 | Berberine on the Secretion of Incretin | / |
| Dyslipidemias\|Dysglycemia | Unknown | 120 | Berberol® K; Placebo | 12 months | 2024-09 | NCT06348550 | Effect of the Association of Berberine Fitosoma® and Monacolin K MonaKoPure® on Glyco-metabolic Parameters in Dysglycemic and Dyslipidemic Patients. | * |
| T2DM (Type 2 Diabetes Mellitus) | Completed | 408 | HTD1801; Placebo | 24 weeks | 2025-08-04 | NCT06350890 | Efficacy and Safety of Berberine Ursodeoxycholate (HTD1801) in Patients With Type 2 Diabetes | ★ |
| Type 2 Diabetes Mellitus | Completed | 551 | HTD1801; Placebo | 24 weeks | 2025-07-30 | NCT06353347 | Efficacy and Safety of Berberine Ursodeoxycholate (HTD1801) in Patients With Type 2 Diabetes Inadequately Controlled With Metformin | ★ |
| Type 2 Diabetes Mellitus | Completed | 113 | HTD1801; Placebo; HTD1801 | 12 weeks | 2023-01-17 | NCT06411275 | A Phase 2 Study of Berberine Ursodeoxycholate (HTD1801) in Patients With Type 2 Diabetes Inadequately Controlled With Diet and Exercise | ★ |
| T2DM (Type 2 Diabetes Mellitus) | Completed | 367 | HTD1801; Dapagliflozin | 24 weeks | 2025-07-25 | NCT06415773 | Effects of Berberine Ursodeoxycholate (HTD1801) Versus Dapagliflozin in Patients With Type 2 Diabetes Inadequately Controlled With Metformin | ★ |
| Diabetes Mellitus\|Obesity | Completed | 50 | Berberine; Gymnema sylvestre | 3 months | 2017-02-01 | NCT06426966 | Gymnema Sylvestre vs Berberine in Obesity Gene Expression of Adipokines | / |
| Diabetes Mellitus | Completed | 40 | Berberine; Berberine | 12 weeks | 2024-10-22 | NCT06863922 | A Pilot Study of Berberine (Soloways ™) in Patients with Type 2 Diabetes Mellitus Carrying TCF7L2 Polymorphisms | / |
| Diabetes Mellitis | Not Yet Recruiting | 60 | Metformin; Berberine | 12 weeks | 2026-03-01 | NCT06911983 | Comparative Efficacy of Metformin and Berberine Among TCF7L2 (rs7903146) TT vs. CC Genotype Carriers With Type 2 Diabetes | * |
| Diabetes-Related Complications | Recruiting | 40 | No intervention | Not reported in uploaded file | 2027-12-31 | NCT06979440 | Study on the Molecular Mechanism of Berberine to Improve Type 2 Diabetes Mellitus Complicated With Depression | / |
| Obesity & Overweight\|Circadian Rhythm Disorders\|Gut Microbiota Modulation\|Prediabetes (Insulin Resistance, Impaired Glucose Tolerance) | Recruiting | 200 | Verum (A); Placebo (B) | Not reported in uploaded file | 2028-02 | NCT07440147 | Effects of Akkermansia Muciniphila and Berberine Supplementation on Insulin Sensitivity in Night-shift Workers | * |
| Nonalcoholic Fatty Liver Disease | Completed | 184 | Life style intervention; pioglitazone; berberine | 16 weeks | 2011-08 | NCT00633282 | Role of Pioglitazone and Berberine in Treatment of Non-Alcoholic Fatty Liver Disease | / |
| Non-Alcoholic Fatty Liver Disease | Completed | 50 | Berberine | 6 weeks | 2019-06-15 | NCT04049396 | Berberine and Non-Alcoholic Fatty Liver Disease (NAFLD) | / |
| Non Alcoholic Fatty Liver\|Obesity | Unknown | 140 | Probiotic; Berberine; Placebo; Probiotc and Berberine | 12 weeks | 2024-06-01 | NCT05523024 | Effect of Probiotics or Berberine in Hepatic Steatosis Markers, Cardiometabolic and Microbiotic Profile in NAFL. | * |
| Obesity\|Obesity, Abdominal\|NAFLD | Completed | 337 | Berberine plus lifestyle intervention; Placebo plus lifestyle intervention | 6 months | 2024-08-09 | NCT05647915 | Effects of Berberine in Reducing Abdominal Visceral Adipose Tissue Among Individuals with Obesity and NAFLD | * |
| Hyperlipidemia\|Endothelial Dysfunction | Completed | 50 | Mixture of Berberine, Policosanol, Red Yeast, Placebo | 6 weeks | 2008-04 | NCT00654459 | Effects of Armolipid Plus on Cholesterol Levels and Endothelial Function | * |
| Hyperlipidemia\|Low-density-lipoprotein-type\|Elevated Triglycerides | Completed | 118 | Armolipid Plus; placebo | Not reported in uploaded file | 2012-10 | NCT01562080 | Combined Effects of Bioactive Compounds in Lipid Profile | * |
| Hyperlipidemia | Unknown | 100 | Non-pharmacologic intervention; Nutraceuticals (Armolipid Plus) | 12 months | 2017-12 | NCT01649986 | Nutraceuticals to Improve Lipid Profile in European Countries | * |
| Dyslipidemias | Terminated | 24 | Berberine;Atorvastatin or Rosuvastatin; Atorvastatin or Rosuvastatin | Not reported in uploaded file | 2015-09 | NCT01697735 | The Therapeutic Effects of Statins and Berberine on the Hyperlipemia | * |
| Hypercholesterolemia | Completed | 60 | nutraceutical combination (red yeast rice, policosanols and berberine) | 24 weeks | 2013-12 | NCT02078167 | Long Term Efficacy and Tolerability of a Nutraceutical Combination (Red Yeast Rice, Policosanols and Berberine) in Low-moderate Risk Hypercholesterolemic Patients: a Double-blind, Placebo Controlled Study | * |
| Atherosclerosis\|Inflammation\|Hypercholesterolemia | Completed | 100 | Nutraceutical Combination; Standard of Care; Placebo | 3 months | 2015-02 | NCT02422927 | Nutraceutical Combination in Patients With Low-grade Systemic Inflammation | * |
| Mixed Dyslipidemia | Completed | 36 | Berberine; Bezafibrate; Berberine plus Bezafibrate | 90 days | 2020-04-01 | NCT02548832 | Bezafibrate Plus Berberine in Mixed Dyslipidemia | * |
| Dyslipidemia | Completed | 63 | Nutraceutical; Control | 3 months | 2018-01-12 | NCT03019263 | Berberine, Chlorogenic Acid and Tocotrienols in Menopause-associated Dyslipidemia | * |
| Coronary Artery Disease\|Percutaneous Coronary Intervention | Recruiting | 64 | Berberine; Standard treatment; Aspirin; Clopidogrel | Not reported in uploaded file | 2026-08 | NCT03378934 | Anti-platelet Effect of Berberine in Patients After Percutaneous Coronary Intervention | / |
| Hypercholesterolemia\|Inflammation\|Atherosclerosis | Completed | 26 | Nutraceutical combination (NC); No nutraceutical combination (noNC) | 3 months | 2017-11-30 | NCT03470376 | NUtraceutical TReatment for hYpercholesterolemia in HIV-infected Patients | * |
| Cardiovascular Risk Factor | Completed | 84 | Berberine; Placebo | 8 weeks | 2020-11-01 | NCT03770325 | A Mechanistic Randomized Controlled Trial on the Cardiovascular Effect of Berberine | / |
| Stable Coronary Artery Disease\|Percutaneous Coronary Intervention | Unknown | 24 | Berberine; Aspirin; Clopidogrel; Statin | Not reported in uploaded file | 2020-12-30 | NCT04434365 | Effect of Berberine for Endothelial Function and Intestinal Microflora in Patients With Coronary Artery Disease | * |
| Hypertension\|Endothelial Dysfunction\|Blood Pressure | Unknown | 30 | berberine hydrochloride; lifestyle intervention group | 3 months | 2022-05-22 | NCT04790942 | Effect of Berberine Hydrochloride on Blood Pressure and Vascular Endothelial Function in Patients With Hypertension | / |
| Dyslipidemias | Unknown | 36 | Berberine-based Food Supplement, Artichoke Extract, Phytosterols, Fenugreek and SelectSIEVE® OptiChol; Berberine-based Food Supplement, Artichoke Extract, Phytosterols, Fenugreek and SelectSIEVE® OptiChol | 12 weeks | 2024-05 | NCT06340308 | Clinical Study With Berberine-based Food Supplement, Artichoke Extract, Phytosterols, Fenugreek and SelectSIEVE® OptiChol | / |
| Hyperlipidemia | Enrolling By Invitation | 100 | Berberine; Placebo | 4 months | 2026-03 | NCT06782646 | Study on the Sex-specific, Lipid- and Lipoprotein-lowering Effects of Berberine | / |
| Polycystic Ovary Syndrome | Unknown | 660 | Letrozole -Berberine; Letrozole; Berberine | 10 weeks | 2013-12 | NCT01116167 | Polycystic Ovary Syndrome (PCOS): Effect Of Letrozole and Berberine | * |
| Polycystic Ovary Syndrome\|Obesity | Unknown | 120 | Berberine; Placebo | 3 months | 2013-06 | NCT01138930 | Effect of Berberine on Hormonal and Metabolic Features in Obese Women With Polycystic Ovary Syndrome (PCOS) | / |
| Polycystic Ovary Syndrome\|Berberine | Completed | 12 | Berberine | 8 weeks | 2021-01-26 | NCT04932070 | Berberine and Polycystic Ovary Syndrome | / |
| Polycystic Ovary Syndrome | Completed | 120 | Berberine | 3 months | 2023-04-15 | NCT05480670 | Treatment Benefits of Berberine Supplementation for Women With PCOS | / |
| Polycystic Ovary Syndrome | Not Yet Recruiting | 150 | Berberine Phytosome (Sophy®); Lifestyle Advice | Not reported in uploaded file | 2027-03-31 | NCT07465575 | Berberine Phytosome for Signs and Symptoms of Polycystic Ovary Syndrome (PCOS) | / |

Note:“*”:BBR-containing combination or nutraceutical; effects may not be attributable to BBR alone.“★”:BBR derivative/conjugate (HTD1801)
